# Supplementary material for: Admissible multiarm stepped‐wedge cluster randomized trial designs
Source: Stat Med. 2018 Nov 6;38(7):1103–19. doi: 10.1002/sim.8022 (PMC6491976; doi:10.1002/sim.8022)
Supplement: Supplementary file 1 — SIM_8022‐Supp‐0001‐sup_material.pdf [file SIM-38-1103-s001.pdf]

Online Supplementary Material for "Admissible multi-arm stepped-wedge cluster randomized trial designs" by Grayling et al.

Optimal cross-sectional designs from Section 3.2

In Supplementary Table 1 we list the optimal designs from Figure 1, discussed in Section 3.2.

D- and A-optimal designs from Section 3.5

Here, in Supplementary Tables 2 and 3, we provide the D- and A-optimal designs discussed in Section 3.5.

Application to binary outcome variables

In this section, we provide a brief description of how our methods can be applied to binary outcome variables (in the case $D = 2$ for a cross-sectional design). Analysing at the cluster level, the following hierarchical model can be utilised for data analysis

$$r_{ij} \sim Bin(m, p_{ij}),$$
$$\text{logit}(p_{ij}) = \mu + \pi_j + \beta_1 X_{ij} + c_i + \epsilon_{ij},$$

where $c_i \sim N(0, \sigma_c^2)$ and $\epsilon_{ij} \sim N(0, \sigma_\epsilon^2/m)$. Moreover, r_{ij} is the number of responses observed in cluster i in period j, and p_{ij} is therefore the probability of response in cluster i in period j.

We can then apply our methodology by assuming that $\sigma_e^2/m = 1/\{m\bar{p}(1-\bar{p})\}$, where $\bar{p}$ is the average response rate. In practice, one would need to then assess the performance of the approximation via a simulation study to assess the empirical power of identified efficient designs. As discussed in Section 4, we may reasonably anticipate that such approximation based results are likely to only be reliable for large sample sizes.

Design 1	Design 2	Design 3	Design 4
$\begin{pmatrix} 0&0&0&0&0&0 \\ 0&0&0&0&0&0 \\ 0&0&0&0&0&0 \\ 0&0&0&0&0&0 \\ 0&0&0&0&1&1 \\ 0&1&1&1&1&1 \\ 1&1&1&1&1&1 \\ 1&1&1&1&1&1 \\ 1&1&1&1&1&1 \\ 1&1&1&1&1&1 \end{pmatrix}$	$\begin{pmatrix} 0&0&0&0&0&0 \\ 0&0&0&0&0&0 \\ 0&0&0&0&0&1 \\ 0&0&0&0&1&1 \\ 0&0&0&1&1&1 \\ 0&0&0&1&1&1 \\ 0&0&1&1&1&1 \\ 0&1&1&1&1&1 \\ 1&1&1&1&1&1 \\ 1&1&1&1&1&1 \end{pmatrix}$	$\begin{pmatrix} 0&0&0&0&0&0 \\ 0&0&0&0&0&1 \\ 0&0&0&0&0&1 \\ 0&0&0&0&1&1 \\ 0&0&0&1&1&1 \\ 0&0&0&1&1&1 \\ 0&0&1&1&1&1 \\ 0&1&1&1&1&1 \\ 0&1&1&1&1&1 \\ 1&1&1&1&1&1 \end{pmatrix}$	$\begin{pmatrix} 0&0&0&0&0&0 \\ 0&0&0&0&0&1 \\ 0&0&0&0&1&1 \\ 0&0&0&0&1&1 \\ 0&0&0&1&1&1 \\ 0&0&0&1&1&1 \\ 0&0&1&1&1&1 \\ 0&1&1&1&1&1 \\ 0&1&1&1&1&1 \\ 1&1&1&1&1&1 \end{pmatrix}$
**Design 5**	**Design 6**	**Design 7**	**Design 8**
$\begin{pmatrix} 0&0&0&0&0&0 \\ 0&0&0&0&0&0 \\ 0&0&0&0&0&0 \\ 0&0&0&0&0&0 \\ 0&0&0&0&0&1 \\ 0&1&1&1&1&1 \\ 1&1&1&1&1&1 \\ 1&1&1&1&1&1 \\ 1&1&1&1&1&1 \\ 1&1&1&1&1&1 \end{pmatrix}$	$\begin{pmatrix} 0&0&0&0&0&0 \\ 0&0&0&0&0&0 \\ 0&0&0&0&0&1 \\ 0&0&0&0&1&1 \\ 0&0&0&1&1&1 \\ 0&0&1&1&1&1 \\ 0&1&1&1&1&1 \\ 1&1&1&1&1&1 \\ 1&1&1&1&1&1 \\ 1&1&1&1&1&1 \end{pmatrix}$	$\begin{pmatrix} 0&0&0&0&0&0 \\ 0&0&0&0&0&1 \\ 0&0&0&0&0&1 \\ 0&0&0&0&1&1 \\ 0&0&0&1&1&1 \\ 0&0&1&1&1&1 \\ 0&0&1&1&1&1 \\ 0&1&1&1&1&1 \\ 1&1&1&1&1&1 \\ 1&1&1&1&1&1 \end{pmatrix}$	$\begin{pmatrix} 0&0&0&0&0&0 \\ 0&0&0&0&0&0 \\ 0&0&0&0&0&0 \\ 0&0&0&0&0&0 \\ 0&0&0&0&0&0 \\ 1&1&1&1&1&1 \\ 1&1&1&1&1&1 \\ 1&1&1&1&1&1 \\ 1&1&1&1&1&1 \\ 1&1&1&1&1&1 \end{pmatrix}$
**Design 9**	**Design 10**	**Design 11**	
$\begin{pmatrix} 0&0&0&0&0&0 \\ 0&0&0&0&0&0 \\ 0&0&0&0&0&0 \\ 0&0&0&0&0&1 \\ 0&0&0&0&1&1 \\ 0&0&1&1&1&1 \\ 0&1&1&1&1&1 \\ 1&1&1&1&1&1 \\ 1&1&1&1&1&1 \\ 1&1&1&1&1&1 \end{pmatrix}$	$\begin{pmatrix} 0&0&0&0&0&0 \\ 0&0&0&0&0&0 \\ 0&0&0&0&0&0 \\ 0&0&0&0&0&1 \\ 0&0&0&0&1&1 \\ 0&0&1&1&1&1 \\ 1&1&1&1&1&1 \\ 1&1&1&1&1&1 \\ 1&1&1&1&1&1 \\ 1&1&1&1&1&1 \end{pmatrix}$	$\begin{pmatrix} 0&0&0&0&0&0 \\ 0&0&0&0&0&0 \\ 0&0&0&0&0&0 \\ 0&0&0&0&0&0 \\ 0&0&0&0&1&1 \\ 0&0&1&1&1&1 \\ 1&1&1&1&1&1 \\ 1&1&1&1&1&1 \\ 1&1&1&1&1&1 \\ 1&1&1&1&1&1 \end{pmatrix}$	

Supplementary Table 1: The optimal designs from Figure 1 are presented.

Factor	D-optimal designs				
$E(\rho)$	$\{0,\ldots,0.10\}$	$\{0.11,\ldots,0.19\}$	$\{0.20,\ldots,0.46\}$	$\{0.47,0.48\}$	0.49
X	$\begin{pmatrix}0&0&0&0&0&0\\0&0&0&0&0&0\\1&1&1&1&1&1\\1&1&1&1&1&1\\2&2&2&2&2&2\\2&2&2&2&2&2\end{pmatrix}$	$\begin{pmatrix}0&0&0&0&0&0\\0&0&0&0&0&1\\0&1&1&1&1&1\\1&1&1&1&1&2\\1&2&2&2&2&2\\2&2&2&2&2&2\end{pmatrix}$	$\begin{pmatrix}0&0&0&0&0&0\\0&0&0&0&1&1\\0&0&1&1&1&1\\1&1&1&1&2&2\\1&1&2&2&2&2\\2&2&2&2&2&2\end{pmatrix}$	$\begin{pmatrix}0&0&0&0&0&1\\0&0&0&0&1&1\\0&0&1&1&1&2\\1&1&1&2&2&2\\1&1&2&2&2&2\\2&2&2&2&2&2\end{pmatrix}$	$\begin{pmatrix}0&0&0&0&0&0\\0&0&0&0&1&1\\0&0&0&1&1&1\\0&1&1&1&2&2\\1&1&2&2&2&2\\1&2&2&2&2&2\end{pmatrix}$
$E(\rho)$	$\{0.50,\ldots,0.53\}$	$\{0.54,0.55,0.56\}$	0.57	$\{0.58,\ldots,0.79\}$	0.80
X	$\begin{pmatrix}0&0&0&0&0&1\\0&0&0&0&1&1\\0&0&1&1&1&2\\1&1&1&2&2&2\\1&1&2&2&2&2\\2&2&2&2&2&2\end{pmatrix}$	$\begin{pmatrix}0&0&0&0&0&0\\0&0&0&0&1&1\\0&0&0&1&1&1\\0&1&1&1&2&2\\1&1&2&2&2&2\\1&2&2&2&2&2\end{pmatrix}$	$\begin{pmatrix}0&0&0&0&0&1\\0&0&0&0&1&1\\0&0&1&1&1&2\\1&1&1&2&2&2\\1&1&2&2&2&2\\2&2&2&2&2&2\end{pmatrix}$	$\begin{pmatrix}0&0&0&0&0&1\\0&0&0&0&1&1\\0&0&0&1&1&2\\0&1&1&2&2&2\\1&1&2&2&2&2\\1&2&2&2&2&2\end{pmatrix}$	$\begin{pmatrix}0&0&0&0&0&1\\0&0&0&0&1&1\\0&0&0&1&1&2\\0&0&1&2&2&2\\1&1&2&2&2&2\\1&2&2&2&2&2\end{pmatrix}$
$E(\rho)$	0.81	0.82	0.83	0.84	0.85
X	$\begin{pmatrix}0&0&0&0&0&1\\0&0&0&0&1&1\\0&0&0&1&2&2\\0&1&1&2&2&2\\1&1&2&2&2&2\\1&2&2&2&2&2\end{pmatrix}$	$\begin{pmatrix}0&0&0&0&0&1\\0&0&0&0&1&1\\0&0&0&1&1&2\\0&0&1&2&2&2\\1&1&2&2&2&2\\1&2&2&2&2&2\end{pmatrix}$	$\begin{pmatrix}0&0&0&0&0&1\\0&0&0&0&1&1\\0&0&0&1&2&2\\0&1&1&2&2&2\\1&1&2&2&2&2\\1&2&2&2&2&2\end{pmatrix}$	$\begin{pmatrix}0&0&0&0&0&1\\0&0&0&0&1&1\\0&0&0&1&1&2\\0&0&1&2&2&2\\1&1&2&2&2&2\\1&2&2&2&2&2\end{pmatrix}$	$\begin{pmatrix}0&0&0&0&0&1\\0&0&0&0&1&1\\0&0&0&1&2&2\\0&0&1&2&2&2\\1&1&2&2&2&2\\1&2&2&2&2&2\end{pmatrix}$
$E(\rho)$	0.86	$\{0.87,0.88,0.89\}$	0.90	$\{0.91,\ldots,0.99\}$	1.00
X	$\begin{pmatrix}0&0&0&0&0&1\\0&0&0&0&1&1\\0&0&0&1&1&2\\0&0&1&2&2&2\\0&1&2&2&2&2\\1&2&2&2&2&2\end{pmatrix}$	$\begin{pmatrix}0&0&0&0&0&1\\0&0&0&0&1&2\\0&0&0&1&2&2\\0&1&1&2&2&2\\1&1&2&2&2&2\\1&2&2&2&2&2\end{pmatrix}$	$\begin{pmatrix}0&0&0&0&0&1\\0&0&0&0&1&1\\0&0&0&1&2&2\\0&0&1&2&2&2\\0&1&2&2&2&2\\1&2&2&2&2&2\end{pmatrix}$	$\begin{pmatrix}0&0&0&0&0&1\\0&0&0&0&1&2\\0&0&0&1&2&2\\0&0&1&2&2&2\\0&1&2&2&2&2\\1&2&2&2&2&2\end{pmatrix}$	$\begin{pmatrix}0&0&0&0&0&0\\0&0&0&1&2&2\\0&0&0&1&2&2\\0&0&0&1&2&2\\0&0&0&1&2&2\\0&0&0&1&2&2\end{pmatrix}$

Supplementary Table 2: D-optimal allocation matrices for cross-sectional designs with $D = 3$. The D-optimal allocation matrices in the case $\mathfrak{I} = \{6\}$, $\mathfrak{C} = \{\mathfrak{C}_6\} = \{6\}$, $\mathfrak{M} = \mathfrak{M}_{6,6} = \{8\}$, and $\sigma^2 = 1$, with $w = 0$ and $\beta = 1$ are shown for $E(\rho) \in \{0, 0.01, \ldots, 1\}$. No restrictions are placed on $\mathfrak{X}$ other than the identifiability of Equation 1. Each allocation matrix was identified via our exhaustive search method.

Factor	A-optimal designs					
$E(\rho)$	$\{0,\ldots,0.06\}$	0.07	$\{0.08,\ldots,0.11\}$	$\{0.12,\ldots,0.30\}$	$\{0.31, 0.32, 0.33\}$	$\{0.34, 0.35\}$
X	$\begin{pmatrix}0&0&0&0&0&0\\0&0&0&0&0&1\\1&1&1&1&1&1\\1&1&1&1&1&1\\1&2&2&2&2&2\\2&2&2&2&2&2\end{pmatrix}$	$\begin{pmatrix}0&0&0&0&0&0\\0&0&0&0&1&1\\1&1&1&1&1&1\\1&1&1&1&1&1\\1&1&2&2&2&2\\2&2&2&2&2&2\end{pmatrix}$	$\begin{pmatrix}0&0&0&0&0&0\\0&0&0&0&1&1\\0&1&1&1&1&1\\1&1&1&1&1&2\\1&1&2&2&2&2\\2&2&2&2&2&2\end{pmatrix}$	$\begin{pmatrix}0&0&0&0&0&0\\0&0&0&0&1&1\\0&0&1&1&1&1\\1&1&1&1&2&2\\1&1&2&2&2&2\\2&2&2&2&2&2\end{pmatrix}$	$\begin{pmatrix}0&0&0&0&0&0\\0&0&0&0&1&1\\0&0&1&1&1&1\\1&1&1&1&2&2\\1&1&2&2&2&2\\1&2&2&2&2&2\end{pmatrix}$	$\begin{pmatrix}0&0&0&0&0&0\\0&0&0&0&1&1\\0&0&1&1&1&1\\0&1&1&1&2&2\\1&1&2&2&2&2\\1&2&2&2&2&2\end{pmatrix}$
$E(\rho)$	$\{0.36,\ldots,0.39\}$	$\{0.40,\ldots,0.62\}$	$\{0.63,\ldots,0.66\}$	0.67	$\{0.68, 0.69\}$	$\{0.70,\ldots,0.81\}$
X	$\begin{pmatrix}0&0&0&0&0&1\\0&0&0&0&1&1\\0&0&1&1&1&2\\1&1&1&1&2&2\\1&1&2&2&2&2\\2&2&2&2&2&2\end{pmatrix}$	$\begin{pmatrix}0&0&0&0&0&1\\0&0&0&0&1&1\\0&0&1&1&1&2\\0&1&1&1&2&2\\1&1&2&2&2&2\\1&2&2&2&2&2\end{pmatrix}$	$\begin{pmatrix}0&0&0&0&0&1\\0&0&0&0&1&1\\0&0&0&1&1&2\\0&1&1&1&2&2\\1&1&2&2&2&2\\1&2&2&2&2&2\end{pmatrix}$	$\begin{pmatrix}0&0&0&0&0&1\\0&0&0&0&1&1\\0&0&1&1&1&2\\0&1&1&2&2&2\\1&1&2&2&2&2\\1&2&2&2&2&2\end{pmatrix}$	$\begin{pmatrix}0&0&0&0&0&1\\0&0&0&0&1&1\\0&0&0&1&1&2\\0&1&1&1&2&2\\1&1&2&2&2&2\\1&2&2&2&2&2\end{pmatrix}$	$\begin{pmatrix}0&0&0&0&0&1\\0&0&0&0&1&1\\0&0&0&1&1&2\\0&1&1&2&2&2\\1&1&2&2&2&2\\1&2&2&2&2&2\end{pmatrix}$
$E(\rho)$	0.82	0.83	$\{0.84, 0.85, 0.86\}$	$\{0.87, 0.88, 0.89\}$	$\{0.90, 0.91, 0.92\}$	0.93
X	$\begin{pmatrix}0&0&0&0&0&1\\0&0&0&0&1&1\\0&0&0&1&1&2\\0&0&1&2&2&2\\1&1&2&2&2&2\\1&2&2&2&2&2\end{pmatrix}$	$\begin{pmatrix}0&0&0&0&0&1\\0&0&0&0&1&1\\0&0&0&1&2&2\\0&1&1&2&2&2\\1&1&2&2&2&2\\1&2&2&2&2&2\end{pmatrix}$	$\begin{pmatrix}0&0&0&0&0&1\\0&0&0&0&1&1\\0&0&0&1&1&2\\0&0&1&2&2&2\\1&1&2&2&2&2\\1&2&2&2&2&2\end{pmatrix}$	$\begin{pmatrix}0&0&0&0&0&1\\0&0&0&0&1&2\\0&0&1&1&2&2\\0&1&1&2&2&2\\1&1&2&2&2&2\\1&2&2&2&2&2\end{pmatrix}$	$\begin{pmatrix}0&0&0&0&0&1\\0&0&0&0&1&1\\0&0&0&1&1&2\\0&0&1&2&2&2\\0&1&2&2&2&2\\1&2&2&2&2&2\end{pmatrix}$	$\begin{pmatrix}0&0&0&0&0&1\\0&0&0&0&1&2\\0&0&0&1&2&2\\0&1&1&2&2&2\\1&1&2&2&2&2\\1&2&2&2&2&2\end{pmatrix}$
$E(\rho)$	0.94	$\{0.95, 0.96\}$	0.97	$\{0.98, 0.99\}$	1.00	
X	$\begin{pmatrix}0&0&0&0&0&1\\0&0&0&0&1&1\\0&0&0&1&1&2\\0&0&1&2&2&2\\0&1&2&2&2&2\\1&2&2&2&2&2\end{pmatrix}$	$\begin{pmatrix}0&0&0&0&0&1\\0&0&0&0&1&1\\0&0&0&1&2&2\\0&0&1&2&2&2\\0&1&2&2&2&2\\1&2&2&2&2&2\end{pmatrix}$	$\begin{pmatrix}0&0&0&0&0&1\\0&0&0&0&1&1\\0&0&0&2&2&2\\0&0&1&1&1&2\\0&1&2&2&2&2\\1&1&1&1&2&2\end{pmatrix}$	$\begin{pmatrix}0&0&0&0&0&1\\0&0&0&0&1&2\\0&0&0&1&2&2\\0&0&1&2&2&2\\0&1&2&2&2&2\\1&2&2&2&2&2\end{pmatrix}$	$\begin{pmatrix}0&0&0&0&0&0\\0&0&0&0&0&0\\0&0&0&0&0&0\\1&2&2&2&2&2\\1&2&2&2&2&2\\1&2&2&2&2&2\end{pmatrix}$	

Supplementary Table 3: A-optimal allocation matrices for cross-sectional designs with $D = 3$. The A-optimal allocation matrices in the case $\mathfrak{I} = \{6\}$, $\mathfrak{C} = \{\mathfrak{C}_6\} = \{6\}$, $\mathfrak{M} = \mathfrak{M}_{6,6} = \{8\}$, and $\sigma^2 = 1$, with $w = 0$ and $\beta = 1$ are shown for $E(\rho) \in \{0, 0.01, \ldots, 1\}$. No restrictions are placed on $\mathfrak{X}$ other than the identifiability of Equation 1. Each allocation matrix was identified via our exhaustive search method.
